# The Evolution of Protein Interaction Networks in Regulatory Proteins

**DOI:** 10.1002/cfg.365

**Published:** 2004-02

**Authors:** Gregory D. Amoutzias, David L. Robertson, Erich Bornberg-Bauer

**Affiliations:** 1 School of Biological Sciences University of Manchester 2.205 Stopford Building Oxford Road Manchester M13 9PT UK; 2 Bioinformatics Group Biology Department University of Münster Schlossplatz 4 D-48149 Germany

## Abstract

Interactions between proteins are essential for intracellular communication. They form complex networks which have become an important source for functional analysis of proteins. Combining phylogenies with network analysis, we investigate the evolutionary history of interaction networks from the bHLH, NR and bZIP transcription-factor families. The bHLH and NR networks show a hub-like structure with varying γ values. Mutation and gene duplication play an important role in adding and removing interactions. We conclude that in several of the protein families that we have studied, networks have primarily arisen by the development of heterodimerizing transcription factors, from an ancestral gene which interacts with any of the newly emerging proteins but also homodimerizes.


**Comparative and Functional Genomics**
*Comp Funct Genom* 2004; **5**: 79–84.
Published online in Wiley InterScience (www.interscience.wiley.com). **DOI:** 10.1002/cfg.365

**Conference Review**

### The evolution of protein interaction networks in regulatory proteins

Gregory D. Amoutzias[1], David L. Robertson[1] and Erich Bornberg-Bauer[2]*

[1] *School of Biological Sciences, University of Manchester, 2.205 Stopford Building, Oxford Road, Manchester M13 9PT, UK*
[2] *Bioinformatics Group, Biology Department, University of Münster, Schlossplatz 4, D-48149 Germany*

**Correspondence to:
Erich Bornberg-Bauer,
Bioinformatics Group, Biology
Department, University of
Münster, Schlossplatz 4,
D-48149 Germany.
E-mail: ebb@uni-muenster.de*

Received: 13 November 2003
Revised: 18 November 2003
Accepted: 25 November 2003

### Abstract

**Interactions between proteins are essential for intracellular communication. They form complex networks which have become an important source for functional analysis of proteins. Combining phylogenies with network analysis, we investigate the evolutionary history of interaction networks from the bHLH, NR and bZIP transcription-factor families. The bHLH and NR networks show a hub-like structure with varying $\gamma$ values. Mutation and gene duplication play an important role in adding and removing interactions. We conclude that in several of the protein families that we have studied, networks have primarily arisen by the development of heterodimerizing transcription factors, from an ancestral gene which interacts with any of the newly emerging proteins but also homodimerizes.** **Copyright © 2004 John Wiley & Sons, Ltd.**

**Keywords:** **protein–protein interaction networks; eukaryotic transcription factors; gene duplication; homo/heterodimerization; small world networks**

### Introduction

Genome, proteome and other 'ome' projects have generated a vast amount of data over the last few years. These data can be analysed by comparative analysis. Proteome analysis became most important for studying regulatory proteins, since many signalling proteins, from membrane-bound receptors down to the DNA-binding regulatory elements (transcription factors), interact directly with each other via so-called protein–protein interactions (PPIs). Some of these interactions are 'unspecific', e.g. both SH3 and SH2 domains interact with many other domains [8,27]. Since some of the interaction partners interact with yet other proteins, this gives rise to a 'network of interactions' where proteins can be imagined as nodes that are connected by edges, representing their physical interactions. However, interactions can also be very specific and limited to only few partner proteins, e.g. many transcription factors heterodimerize with several partners under specific physiological conditions and expression states, while they may homodimerize under other conditions. An example is the leucine zipper-mediated interaction, e.g. the competing Jun–Fos/Jun–Jun interactions in the bZIP family [7,19] or the much weaker Mad–Max/Max–Max interactions in the bHLH family [7,12,17].

The molecular details of dimerization are very complicated and are not the focus of this study. Several groups have begun to investigate the 'global' features of PPI networks, trying to infer functional properties by applying statistical methods to the analysis of the networks [9,14,24,25]. Among the most intriguing of these findings are their small-world characteristics. Although the network is very big, each protein is linked to every other by chains of only a few edges. This is possible because some proteins interact with many others, representing so-called 'hubs', and many have only very few interactions [14]. Furthermore, these interactions appear to be confined to cellular compartments [24].

Copyright © 2004 John Wiley & Sons, Ltd.

The reliability of experimental PPI data is controversially discussed [13,22,23]. However, one should consider that binding is not an all-or-nothing process and at least *in vivo* interactions are frequently competitive, as shown in the bZIP network [18]. Furthermore, the combination of data from various sources can significantly improve their quality. In our research efforts we have collated database information with data mined from the literature to generate more reliable, 'confirmed', datasets [1]. Several models of network evolution have been developed recently [20,21,26,29]. Some models are based on gene duplication, some on domain rearrangements. Others assume that an existing initial network is duplicated when all genes coding for the interacting proteins are duplicated simultaneously. This could happen, for example, via a whole-genome duplication or other large-scale duplication events, such as tandem duplications. In the following a certain fraction of interactions is assumed to be lost again.

The goal of this study is to complement existing perspectives on network evolution with studies based on phylogenies and comparative analysis from genomic and proteomic data. We have chosen to work on several families of eukaryotic transcription factors for which many data from different sources are known and for which phylogenies are either known or can be computed with a reasonable reliability. In particular, we concentrate on the question of how the evolution of interaction specificity, such as homo- vs. heterodimerization, may reveal the evolutionary dynamics of network evolution. Accordingly, results on the families of NR, bZIP and bHLH proteins are reported and discussed in the following.

### Methods

Interactions for all three networks were extracted from a literature search in PubMed, with the focus on mammalian transcription factors (**http://www4.ncbi.nlm.nih.gov/PubMed/**). Specifically for the bZIPs, interactions were extracted from Newman and Keating [18], in which protein arrays were used to test 492 pairings of a nearly complete set of coiled-coil strands from all known human bZIP proteins. We included interactions that were symmetrical in the consensus interaction matrix, meaning that the use of the protein in the array surface or as a probe was not inhibiting the interaction. Also, we disregarded interactions with $Z < 1$, which is the threshold value for the signal : noise ratio. Data for bHLH proteins are the same as in Amoutzias *et al.* [1] but with close family members collapsed to one node. For the NR family we excluded all data for which interactions were shown not to be direct.

### Results

### bHLH proteins

We first focus on bHLH proteins. They represent an ancient family of transcription factors being present in all eukaryotic clades, that expanded since the emergence of multicellularity [16]. bHLH proteins are involved in cell-cycle regulation, metabolic sensing and tissue-specific development. Their main constituent is a ≈60 amino acid-long domain comprising the DNA-binding basic region and the HLH motif which mediates interaction. However, they usually also contain additional dimerization domains which contribute to the specification of homo- and heterodimerization. While further details on their evolution are reported elsewhere [4,16], the feature which is most relevant for this study is that five major groups exist. These can be distinguished by their domain architecture, in particular the presence of the additional dimerization domain (leucine zipper — LZ, PAS, Orange), such that their clustering into groups is fairly reliable. Recently, we found that PPIs between bHLH proteins form two hub-based networks. One of them can be divided into two hub-based subnetworks. The two networks have a striking similarity in their topology but no interactions between any member of one family to any member of the other family are known to exist [1].

Analysing these networks of protein families (Figure 1), the most prominent feature appeared to be that, just like the overall network, they have a hub-like topology. Interaction data have been obtained from a number of different sources and the low number of interactions for the majority of the nodes has been explicitely confirmed by experiments on these nodes. Hubs are a feature of scale-free networks and such properties have been observed in social networks, the world-wide web, the western US power grid, citations of scientific

Copyright © 2004 John Wiley & Sons, Ltd. 

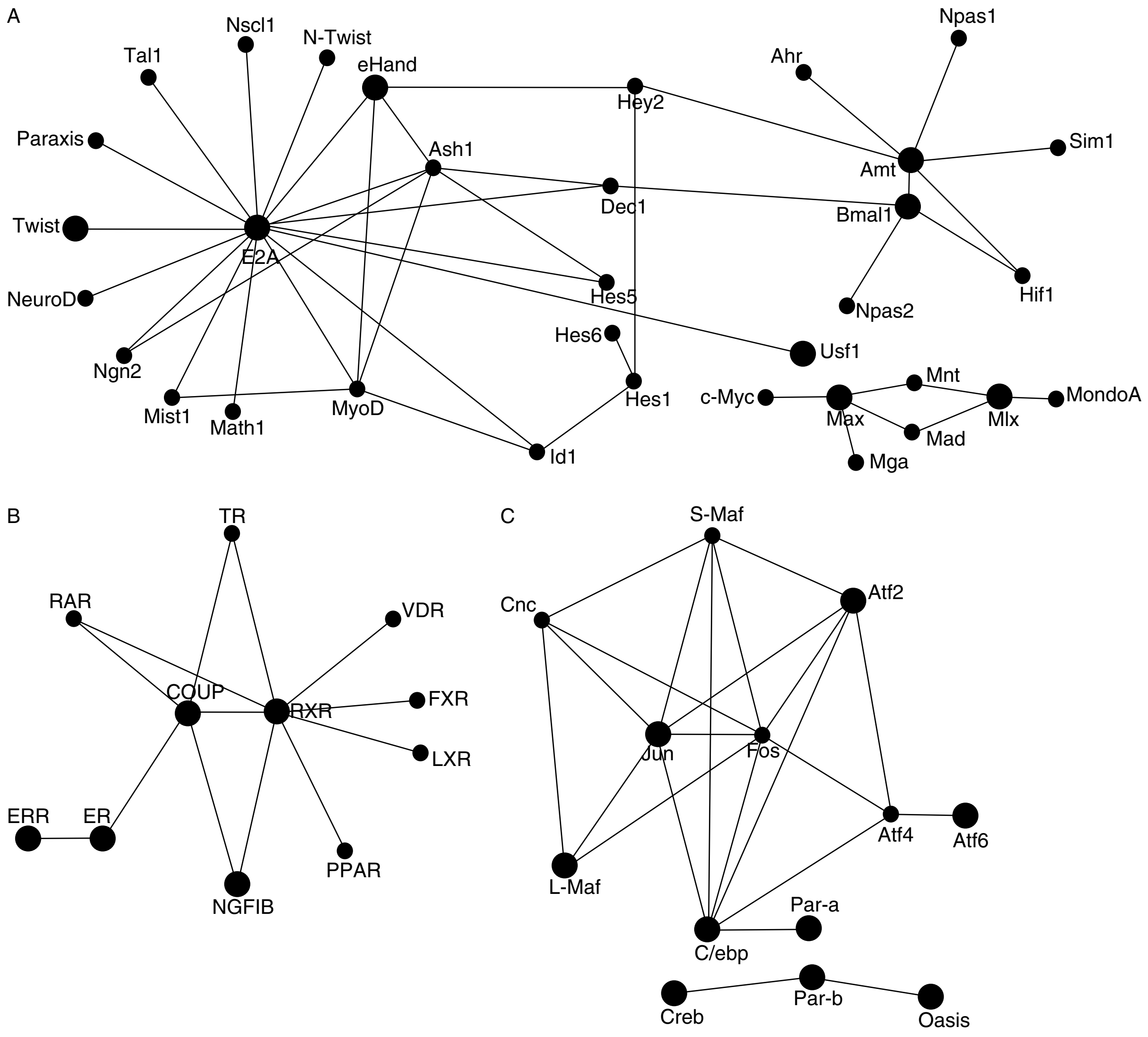

**Figure 1.** Protein interaction networks of eukaryotic transcription factors: bHLH (A), NR (B) and bZIP (C). Homodimerization is indicated by larger dots publications, metabolic networks, protein domains, protein interaction networks and the distribution of proteins in sequence space [3,5,6,31,32]. For the bHLH protein-interaction network, we calculated the frequency of nodes with $K$ interactions, $P(K)$, and plotted this against the number of interactions, $K$.

The plot (Figure 2.) of $ln[P(K)] - ln(K)$ shows clearly that the bHLH PPI network is scale-free, because the distribution of connectivity decays as a power-law $P(K) = K^{-\gamma}$, where $\gamma \approx 1$. A scale-free network is a non-homogeneous network with a few highly connected nodes (the hubs) and many poorly connected nodes (the peripheral members). Other analyses on scale-free networks estimated that $\gamma$ is usually in the range 2–3 [11]. The biological significance of this lower value for $\gamma$ appears to be a direct consequence of the fact that gene duplication events (single or large-scale) have generated new peripheral proteins that then interact preferentially with the hub. Apparently the homodimerizing factors are the most highly linked (at least within their networks) and represent hubs (Figure 2). Consequently, we conclude that homodimerization was the ancestral function. This feature was maintained with the emergent hub, even though the peripheral

Copyright © 2004 John Wiley & Sons, Ltd.

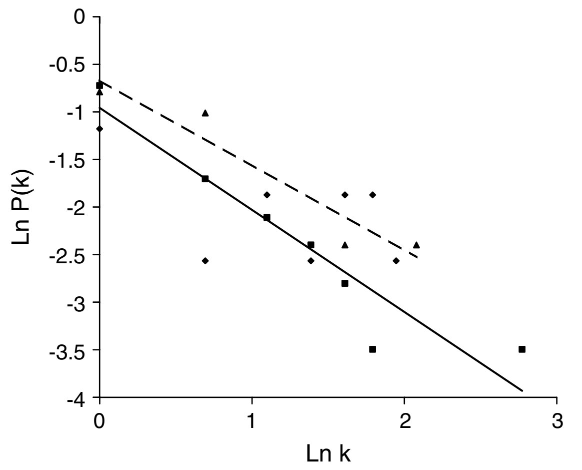

**Figure 2.** Scaling behaviour of protein interaction networks of eukaryotic transcription factors: bHLH (squares), NR (triangles), bZIP (diamonds). Linear fits for bHLH (full line, $\gamma \approx 1$) and NR (dashed line, $\gamma \approx 0.95$)

members, which emerged as constitutive interaction partners, became free to bind only under more limited physiological conditions. This is reflected by the fact that hubs are typically constitutive and widely expressed, while peripheral members are often tissue-specific. It is also noteworthy that in a recently published model [20], which is based on gene duplication alone, $\gamma \approx 1.2$ when only few links (PPIs) are lost, while the 'classical' values of $\gamma \approx 2-3$ require a relatively high rate of loss.

### NR proteins

Members from the superfamily of nuclear receptor proteins are transcription factors which can homo- or heterodimerize or even bind to DNA as monomers. With the exception of a few so-called 'orphan receptors', they are activated by binding a ligand and regulate metabolic pathways, development homoeostasis and reproduction [15].

NR proteins are organized in four domains: the N-terminal transactivation domain A, the DNA-binding domain (DBD) that contains two zinc-fingers, the ligand-binding domain (LBD) and a flexible hinge between the DBD and the LBD. The DBD and the LBD are involved in dimerization.

Phylogenies based on sequence analysis between LBD and DBD revealed six distinct subfamilies (I–VI), with two of them (I and IV) being more closely related than the rest [15]. No other phylogenetic relationship between the subfamilies has been reliably inferred as yet.

Again, the interaction pattern is well correlated with the group membership. While there are, in general, no interactions between different groups of receptors within subfamilies I and IV, most members of subfamilies I and IV tend to form efficient heterodimers with some members of subfamily II, whereas in other subfamilies, homodimerization is most frequent (see Figure 1).

Laudet [15] and co-workers investigated the evolutionary rates for the members of the NR family. While they reported strong differences in evolutionary rates between individual proteins, there were no significant differences among the six subfamilies. However, by relating individual proteins with protein–protein interaction data it becomes apparent that it is the hubs, which are generally also homodimerizing, that evolved slower than the peripheral members. This is in agreement with the idea that hubs are the predecessors from which new interaction partners (repressors or activators) emerged by gene duplication, followed by mutation. The two weakly linked proteins ER and ERR (see Figure 1b) are homodimerizing. Apparently they arose more recently and have not as yet differentiated into an independent network. Further evidence that the hubs are the ancestral part of the network comes from the fact that they were present in early metazoans such as sponges and cnidariaus, whereas the peripheral members that belong to subfamilies I and IV appeared much later, after the emergence of the Bilateria [10,30].

### bZIP proteins bZIP proteins are an ancient family of transcription factors present in all eukaryotic clades. They regulate genes that are involved in proliferation, immune response, cell death and response to stress and toxicity [2]. bZIP proteins are named after their well-conserved $\alpha$-helical bZIP domain. The bZIP domain comprises the DNA-binding basic region (BR) and, C-terminally adjacent to it, the leucine zipper (LZ), which forms a coiled coil and determines the dimerization partner for homo- and heterodimers [7,17–19].

It is difficult to reconstruct the phylogeny from sequence information alone and domain arrangements are not as conclusive as for the bHLH family.

Copyright © 2004 John Wiley & Sons, Ltd. 

However, a classification has been suggested, based on the amino acid composition and on dimerization partners [28]. More comprehensive interaction data from bZIP proteins have been analysed most recently, using protein chip technology [18]. Applying network analysis as above reveals a more even distribution of connectivities; however, there is still a fair amount of clustering (Figure 2). This is an indication of an evolutionary mechanism for this family which is different from the bHLH and NR families. Also, there is no such clear differentiation between homo- and heterodimerization, since most proteins have at least a limited capacity to homodimerize.

### Discussion

In this study we have combined network analysis and phylogenies to investigate the emergence of new interactions in the gene networks of eukaryotic transcription factors.

In all three families we have studied (bHLH, bZIP and NR), there is an indication that homodimerization preceded the development of heterodimerization. In NRs, strong evidence comes from phylogenetic studies and the distribution of NR families in early metazoans [10,30]. The evolution of the bHLH networks is also consistent with the ancestral nature of homodimerization. Typically, the ability for homodimerization appears to be conserved, such that hubs emerge from the ancestral homodimerizing proteins. Subsequent gene duplication (large- or small-scale) and mutation results in changes in dimerization properties, thus forming a complex network. In particular, the bHLH family have apparently evolved by repeated single-gene duplications which led to the initial network topology [1]. Subsequent large-scale gene duplications may have increased the complexity of the bHLH network. While the role of gene duplication was also important in the evolution of the NR and bZIP networks, the central role of single gene duplication cannot be confirmed with current data.

The basic principles, i.e. the hub-like structure of the interaction networks, comply with the global features as they were shown by other groups. However, the statistical properties ($\gamma$) for the subnetworks differ between the families and deviate more or less from the global properties of PPI networks as they have been analysed previously. Obviously, these differences reflect different evolutionary dynamics, such as the relative frequency of gene duplication, large-scale duplication events and loss of interactions. The loss of interactions appears to be particularly important in the initial stages of network development and its influence on the value of $\gamma$ appears to be in good agreement with the predictions by Pastor-Satorras and co-workers [20].

Our results have obvious implications for the understanding of network evolution. Further theoretical studies and models of network evolution should consider these variations in $\gamma$ and the fact that, at least in many cases, heterodimerization emerges from homodimerization.

### Acknowledgements

GA is the recipient of a CASE studentship from the EPSRC and AstraZeneca plc. EBB acknowledges support through an MRC international recruitment grant. Thanks to Chris Lockwood, Michaela Falb and Anna Divoli for helpful discussions. GA gratefully acknowledges support by Dimitris and Vasiliki Amoutzias.

### References

1. Amoutzias GD, Robertson DL, Oliver SG, Bornberg-Bauer E. 2003. Convergent evolution of gene networks by single-gene duplication in higher eukaryotes (*submitted*). Supplementary data on: **www.uni-muenster.de/Biologie.Botanik/ebb/bHLH/**.
2. Angel P, Karin M. 1991. The role of Jun, Fos and the AP-1 complex in cell proliferation and transformation. *Biochim Biophys Acta* **1072**: 129–157.
3. Apic G, Gough J, Teichmann SA. 2001. Domain combinations in archaeal, eubacterial and eukaryotic proteomes. *J Mol Biol* **310**: 311–324.
4. Atchley WR, Fitch WM. 1997. A natural classification of the basic helix–loop–helix class of transcription factors. *Proc Natl Acad Sci USA* **94**: 5172–5176.
5. Barabasi A. 2002. *Linked: The New Science of Networks*. Perseus Press: Oxford.
6. Bornberg-Bauer E. 2002. Randomness, structural uniqueness, modularity and neutral evolution in sequence space of model proteins. *Z Phys Chem* **216**: 139–154.
7. Bornberg-Bauer E, Rivals E, Vingron M. 1998. Computational approaches to identify leucine zippers. *Nucleic Acids Res* **26**: 2740–2746.
8. Brannetti B, Via A, Cestra G, Cesareni G, Helmer-Citterich M. 2000. SH3-SPOT: an algorithm to predict preferred ligands to different members of the SH3 gene family. *J Mol Biol* **298**: 313–328.
9. Bu DB, Zhao Y, Cai L, *et al.* 2003. Topological structure analysis of the protein–protein interaction network in budding yeast. *Nucleic Acids Res* **31**: 2443–2450.

Copyright © 2004 John Wiley & Sons, Ltd. 

10. Escriva H, Safi R, Hanni C, *et al*. 1997. Ligand binding was acquired during evolution of nuclear receptors. *Proc Natl Acad Sci USA* **94**: 6803–6808.
11. Goh KI, Oh E, Jeong H, Khang B, Kim D. 2002. Classification of scale-free networks. *Proc Natl Acad Sci USA* **99**: 12 583–12 588.
12. Hurst H. 1994. bZIP proteins. *Protein Profile* **2**: 105–106.
13. Ito T, Ota K, Kubota H, *et al*. 2002. Roles for the two-hybrid system in exploration of the yeast protein interactome. *Mol Cell Proteom* **1**: 561–566.
14. Jeong H, Mason SP, Barabasi A, Oltvai Z. 2001. Lethality and centrality in protein networks. *Nature* **411**: 41–42.
15. Laudet V. 1997. Evolution of the nuclear receptor superfamily: early diversification from an ancestral orphan receptor. *J Mol Endocrinol* **19**: 207–226.
16. Ledent V, Vervoot M. 2001. The basic helix–loop–helix protein family: comparative genomics and phylogenetics analysis. *Genome Res* **11**: 754–770.
17. Muhle-Goll C, Nilges M, Pastore A. 1995. The leucine zippers of the HLH-LZ proteins max and c-myc preferentially form heterodimers. *Biochemistry* **34**: 13 554–13 564.
18. Newman JR, Keating AE. 2003. Comprehensive identification of human bzip interactions with coiled-coil arrays. *Science* **300**: 2097–2101.
19. O'Shea EK, Rutkowski R, Kim PS. 1992. Mechanism of specificity in the fos-jun oncoprotein heterodimer. *Cell* **68**: 699–708.
20. Pastor-Satorras R, Smith E, Sole RV. 2002. Evolving protein interaction networks through gene duplication. *J Theoret Biol* **222**: 199–210.
21. Rzhetsky A, Gomez SM. 2001. Birth of scale-free molecular networks and the number of distinct DNA and protein domains per genome. *Bioinformatics* **17**: 988–996.
22. Salwinski L, Eisenberg D. 2003. Computational methods of analysis of protein–protein interactions. *Curr Opin Struct Biol* **13**: 377–382.
23. Schachter V. 2002. Bioinformatics of large-scale protein interaction networks. *Biotechniques* **S16**: 16–18.
24. Schwikowski B, Uetz P, Fields S. 2000. A network of protein–protein interactions in yeast. *Nature Biotechnol* **18**: 1257–1261.
25. Vazquez A, Flammini A, Maritan A, Vespignani A. 2003a. Global protein function prediction from protein–protein interaction networks. *Nature Biotechnol* **21**: 697–700.
26. Vazquez A, Flammini A, Maritan A, Vespignani A. 2003b. Modeling of protein interaction networks. *ComPlexUs* **1**: 38–44.
27. Villanneva J, Fernandez-Ballester G Querol E, Aviles FX, Serrano L. 2003. Lig and screening by exoproteolysis and mass spectrometry in combination with computer modelling. *J Mol Biol* **330**: 1039–1048.
28. Vinson C, Myakishev M, Acharya S, *et al*. 2002. Classification of human b-zip proteins based on dimerization properties. *Mol Cell Biol* **22**: 6321–6335.
29. Wagner A. 2003. How the global structure of protein interaction networks evolves. *Proc R Soc Lond B* **270**: 457–466.
30. Wiens M, Batel R, Korzhev M, Muller W. 2003. Retinoid x receptor and retinoic acid response in the marine sponge *Suberites domuncula*. *J Exp Biol* **206**: 3261–3271.
31. Wolf YI, Karev G, Koonin EV. 2002. Scale-free networks in biology: new insights into the fundamentals of evolution. *Bioessays* **24**: 105–109.
32. Wuchty S. 2001. Scale-free behaviour in protein domain network. *Mol Biol Evol* **18**: 1694–1702.

Copyright © 2004 John Wiley & Sons, Ltd. 
